# Molecular Level Insights Into the Structural and Dynamic Factors Driving Cytokine Function

**DOI:** 10.3389/fmolb.2021.773252

**Published:** 2021-10-25

**Authors:** Jennifer Y. Cui, George P. Lisi

**Affiliations:** Department of Molecular Biology, Cell Biology and Biochemistry, Brown University, Providence, RI, United States

**Keywords:** cytokine, protein dynamics, immunology, spectroscopy, allostery

## Abstract

Cytokines are key mediators of cellular communication and regulators of biological advents. The timing, quantity and localization of cytokines are key features in producing specific biological outcomes, and thus have been thoroughly studied and reviewed while continuing to be a focus of the cytokine biology community. Due to the complexity of cellular signaling and multitude of factors that can affect signaling outcomes, systemic level studies of cytokines are ongoing. Despite their small size, cytokines can exhibit structurally promiscuous and dynamic behavior that plays an equally important role in biological activity. In this review using case studies, we highlight the recent insight gained from observing cytokines through a molecular lens and how this may complement a system-level understanding of cytokine biology, explain diversity of downstream signaling events, and inform therapeutic and experimental development.

## Introduction

Cytokines are small signaling proteins that serve as effectors of immunity and require spatial or temporal expression to regulate biological outcomes (Altan-Bonnet and Mukherjee, 2019). Cytokines are especially prevalent in disease states where their presence can indicate pathological progression or conversely, a protective effect. For example, signaling cascades that maintain homeostatic balance use cytokines to coordinate appropriate responses that initiate or terminate inflammation. The lens through which researchers often view cytokine biochemistry involves their localization and quantity, using phenomenological models to derive system-level explanations of function (Altan-Bonnet and Mukherjee, 2019). Despite the utility of this approach, the molecular aspects of cytokine function remain unclear, hindering efforts to intuitively modulate their mechanisms and promiscuous interactions. Understanding the structural and environmental factors that stimulate transformations of cytokines is critical to disentangling their role in biological events. Such insight can inform 1) the role played by cytokines as agents of cellular communication, 2) the improvement of tools leveraged for research, and 3) the development of cytokines as therapeutics or targets in clinical treatment plans. In the past decade, many enzymes and metabolic proteins that accommodate multiple non-overlapping functions by “moonlighting” have been shown to utilize intrinsic protein dynamics as a mechanistic driving force (Mani et al., 2015; Boukouris et al., 2016; Chen et al., 2018; Chen et al., 2021). However, structural plasticity in cytokines with broadly demonstrated abilities to harbor multiple functions within compact scaffolds has not been as thoroughly investigated as a moonlighting mechanism.

Researchers use cytokines to replicate immunological conditions in the laboratory in order to study behaviors of specific cell types (Schluns and Lefrançois, 2003; Arango Duque and Descoteaux, 2014; Roan et al., 2019), to replicate extracellular environments, to observe biological responses *in vitro* (Kämpfer et al., 2017; Berghaus et al., 2018; Barnes and Somerville, 2020), and as a measurable biomarker of disease. Cytokines are also used as therapeutics in clinical settings to specifically stimulate immune responses, including interleukins (ILs) in cancer and colony-stimulating factors (CSFs) in general neutropenia (Mehta et al., 2015; Berraondo et al., 2019; Conlon et al., 2019). Given the breadth of the roles played by cytokines intrinsically and as research tools or in clinical settings (i.e*.* from bench to bedside), it is clear that having thorough understanding of the mechanisms driving cytokine function is crucial. One factor that complicates structure-function investigations of cytokines, and the establishment of general rules that govern their function, is the fact that these proteins exert a great deal of their influence under conditions of disease, which are more difficult to mimic in structural characterizations. In this Review, we discuss current efforts to define the mechanisms of cytokines at the “residue level,” where changes in atomic structure or conformational dynamics of the protein yields information relevant to physiological scenarios. We highlight case studies of cytokines with emerging diverse roles, while also showcasing experimental and theoretical approaches for understanding cytokine biochemistry. Though we cannot comprehensively discuss every avenue of investigation in this Review (Künze et al., 2014; Herring et al., 2015; Joseph et al., 2015; Künze et al., 2016; Park et al., 2017; Azimzadeh Irani and Ejtehadi, 2019; Penk et al., 2019; Gangele et al., 2020; Roy, 2020; Yang, 2020), each of the case studies below offers a useful piece insight into an extremely complex class of biomolecules that may aid in the development of broadly applicable strategies to study molecular interactions.

## Case Study I: Macrophage Migration Inhibitory Factor

Macrophage migration inhibitory factor (MIF) is implicated in a wide range of disease states, where elevated MIF levels are markers for tumorigenesis (Bach et al., 2008; Noe and Mitchell, 2020), sepsis (Toldi et al., 2021), acute respiratory distress syndrome (ARDS) (Donnelly et al., 1997; Lai et al., 2003), arthritis (Onodera et al., 1999; Kim et al., 2007; Llamas-Covarrubias et al., 2012), colitis (de Jong et al., 2001), and Malaria (Han et al., 2010; Sun et al., 2012; Baeza Garcia et al., 2018). MIF is highly conserved in diverse organisms and is heavily involved in inflammatory responses, cell cycle control, the sensing of pathogenic stimuli, activation of innate defense responses, recruitment of immune cells, and prevention of p53-mediated apoptosis (Hudson et al., 1999; Mitchell et al., 2002). Despite a compact structural scaffold and a lack of clear modular domains, MIF contains several sequence motifs, including two enzymatic sites for disparate tautomerase and oxidoreductase activities (a third nuclease activity was recently proposed), that provide MIF with a surprising functional complexity (Kleemann et al., 1998; Lubetsky et al., 1999; Wang et al., 2021). MIF is also remarkably amenable to interactions with a number of target proteins, despite its overall architectural rigidity (Mühlhahn et al., 1996), and is proposed to be involved, directly or indirectly, in 49 signaling events. (Subbannayya et al., 2016). This functional promiscuity necessitates an intimate understanding of the mechanism(s) that govern its cellular interactions, but also complicate efforts to assign any singular function to MIF with a high level of mechanistic detail.

**FIGURE 1:**
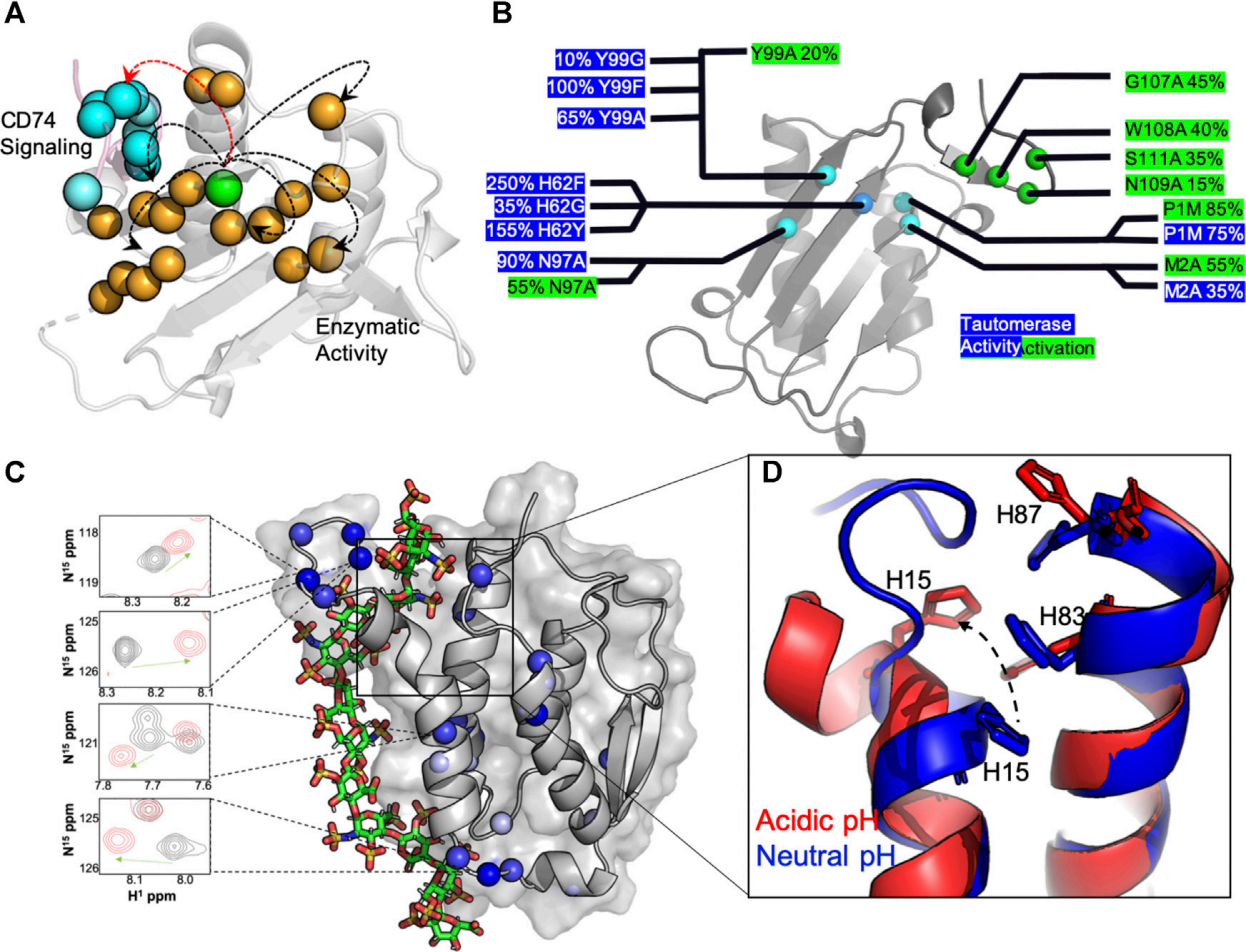
Molecular insights from structure-function studies of MIF and GMCSF. **(A)** Tyr99 mutations (green) induce widespread NMR chemical shifts and line broadening (orange spheres), suggesting that are large portion of the MIF structure “senses” short and long-range correlations (arrows) that influence MIF signaling. The C-terminus of the adjacent MIF monomer, the site of CD74 activation, is shown in pink with blue spheres. **(B)** Experimental measurements of MIF activities determined from mutagenesis. Activity levels are presented as a percentage of wild-type (wt) MIF activity, with enzymatic catalysis (blue) determined from the enol-keto tautomerization of 4-hydroxyphenyl pyruvate (4-HPP) (Lubetsky et al., 1999) and CD74 activation (green) determined from the recruitment of neutrophils to murine lungs *in vivo* (Takahashi et al., 2009). **(C)** A computationally-derived GMCSF structure in the presence of a heparin oligo at pH 5.5. Experimental NMR chemical shift perturbations (blue spheres) are mapped onto GMCSF, and NMR resonances in the presence (red) and absence (black) of heparin are shown to correlate with the proposed binding site. D) An overlay of GMCSF structures at pH 7.4 (blue, PDB: 2GMF) and pH 5.5 (red, from simulations), where disruption of H15, H83 and H87 pi-stacking alignment is clear at acidic pH and in the presence of heparin.

Efforts to understand MIF at the molecular level have focused on 1) studies of mutations that affect its structure and function, 2) studies of signaling within its homotrimeric structure, and 3) studies of interactions between MIF and drug-like molecules (Lubetsky et al., 1999; Mühlhahn et al., 1996; Pantouris et al., 2015; Pantouris et al., 2020; Fan et al., 2013; Trivedi-Parmar and Jorgensen, 2018; Cournia et al., 2009; Cho et al., 2010; Takahashi et al., 2009). Site-directed mutagenesis has been employed by several laboratories to examine the structural and biological impact of perturbations to the MIF catalytic and receptor binding sites (Pantouris et al., 2015; Pantouris et al., 2020; Fan et al., 2013; El-Turk et al., 2012; Oda et al., 2008; El-Turk et al., 2008; Bendrat et al., 1997). X-ray crystallographic reports of >100 MIF structures in the Protein Data Bank show essentially no observable changes in response to mutations, substrates or inhibitors, suggesting MIF is highly rigid and resistant to architectural deformation. However, identical solution NMR experiments show that atomic level fingerprints of MIF variants are unique, indicating that the local structure of MIF is in fact quite flexible in solution and may play a significant role in modulating its biochemistry (Pantouris et al., 2018; Schinagl et al., 2018). More recent work has leveraged these findings to explore the existence of signaling pathways that propagate chemical information between distant regions of the MIF structure. Pantouris, et al characterized two such pathways in successive reports, first demonstrating through molecular dynamics (MD) simulations that nanosecond timescale conformational fluctuations were critical to the organization of the binding interface for the MIF-induced activation of CD74 (Pantouris et al., 2015). Further analysis of correlated dynamics in MIF revealed the CD74 activation signal to originate from a previously uncharacterized residue, Tyr99, at the solvent channel formed by the three-fold symmetric MIF structure (Figure 1A). Interestingly, this allosteric site modulates the dynamics of the MIF protein on multiple timescales when mutated, and not only affects CD74 activation *in vivo*, but also regulates tautomerase enzymatic activity (Figure 1B) through a different signaling pathway originating from Tyr99 (Pantouris et al., 2018; Pantouris et al., 2020; Parkins et al., 2021). The factors that influence the ramping up or down of MIF enzymatic activity include protein dynamics, intramolecular hydrogen bonds and hydrogen bonds with solvent molecules, and pi-stacking interactions from aromatic side chains lining the signaling pathway. At present, it is still unclear how, or if, CD74 activation or tautomerase activity are preferentially activated in the cell. Nonetheless, recent solution studies of MIF revealed two of its biochemical functions to be connected by a region of its structure previously uncharacterized at the molecular level. Numerous studies of MIF in complex with drugs and other modulators have never targeted the Tyr99 regulatory site, but have modulated the same enzymatic and CD74 activities from different locations within MIF, (Cournia et al., 2009; Cho et al., 2010; Trivedi-Parmar and Jorgensen, 2018), presenting a new avenue for structure-function evaluations.

## Case Study II: Granulocyte Macrophage Colony-Stimulating Factor

Granulocyte macrophage colony-stimulating factor (GMCSF) is involved in myelopoiesis (Becher et al., 2016; Ushach and Zlotnik, 2016), immunomodulation (Lotfi et al., 2019), and pro-inflammatory signaling (Bhattacharya et al., 2015; Hamilton, 2019), specifically the promotion of alveolar macrophage activity leading to surfactant accumulation in the lungs (Antoniu, 2010; Kumar et al., 2018). GMCSF has been shown to aggravate conditions such as rheumatoid arthritis and multiple sclerosis (van Nieuwenhuijze et al., 2013; Shiomi et al., 2016), but it can also ameliorate diseases such as type-I diabetes (Frydrych et al., 2019), and is used clinically to combat neutropenia (Mehta et al., 2015). As such, it is a clear example of a cytokine that performs both beneficial and pathological functions. Interestingly, several studies have identified alternate receptor conformations of stoichiometric assembly that can lead to receptor activation (i.e. cell survival, activation, or differentiation) (Hansen et al., 2008; Broughton et al., 2016) and beyond its receptor structure, GMCSF itself has been shown to adopt different structural and dynamic properties dependent on the pH of its chemical environment, which may contribute to its modular signaling or suggest additional non-overlapping roles for GMCSF (Cui et al., 2020).

The GMCSF structure has been studied by X-ray crystallography (Rozwarski et al., 1996), and more recently NMR spectroscopy (Aubin et al., 2008), providing unique insight into its overall fold and plasticity. One particular aspect of GMCSF function that has benefitted substantially from atomic level studies has been the knowledge of its interactions with clinically relevant molecules. In these cases, there has been particular interest in understanding how GMCSF, a clinically administered protein with a promiscuous interactome, could interfere with other therapeutics under conditions of disease. For example, Wettreich and coworkers showed GMCSF to form pH-dependent complexes with heparin, another widely used therapeutic, via analytical chromatography and light scattering (Wettreich et al., 1999). These experiments proposed a binding mechanism and laid the groundwork for understanding how signaling proteins like GMCSF can be tuned by environmental stimuli to form selective ligand complexes. The GMCSF-heparin interaction has since been resolved at the atomic level with NMR spectroscopy under varied solution pH conditions and in the presence of precisely controlled heparin oligosaccharide chain lengths (Cui et al., 2020). The GMCSF protein was found to undergo pH-dependent structural and dynamic changes that modulated its affinity for heparin. Such a pH shift would occur at air-lung interfaces or in tumor microenvironments and was proposed to be essential for the ionization of three histidine residues that nucleate the heparin and the stimulation of multi-timescale dynamics that shift the conformation of N-terminal α-helices to expose the binding pocket. NMR chemical shifts mapped the heparin binding site and relaxation experiments, supported by molecular simulations, confirmed a high level of conformational flexibility along the binding interface and proposed a model for the complex structure (Figure 1C). This binding interaction is dependent not only on the accessibility of the binding pocket, but the size of the heparin as well, where heparins able to span the anchor points at the histidine triad (Figure 1D) and the adjacent flexible linker with several lysine residues bind with greater affinity than smaller chain heparins.

Atomic level studies of GMCSF mapped a clinically-relevant interaction to reveal the binding site and the biophysical factors that influence its accessibility to ligands. This work also reinforced an important aspect of cytokine biochemistry that is intimately tied to the hypothesized dynamic mechanisms of action; the utility of conducting biophysical studies in regimes beyond those of “ideal” conditions. In this case, if GMCSF were only characterized based on its presence in a certain niche (i.e*.* a microenvironment at neutral pH mimicking only circulating GMCSF), the resulting biochemical outcomes would not have captured its high affinity state for heparin and a gap in our understanding of its mechanism(s) would remain. Put another way, if GMCSF is expressed in high quantities in acidic environments and in the presence of high heparin concentrations, a measurable amount of biological activity presumed to be related to the GMCSF concentration would likely not correlate with its expected activity-to-quantity ratio.

## Case Study III: Interleukin-1

Interleukins are a diverse class of cytokines with low sequence identity that are secreted by several cell types (beyond the originally presumed leukocyte-specific secretion that spawned their name). One subclass, the interleukin (IL)-1 family, comprises 11 pro- and anti-inflammatory cytokines that have abnormal expression profiles in many autoimmune diseases (Dinarello, 2018), which presumably affects their signal transduction by the IL-1 receptor (IL-1R). Interestingly, Ge et al recently demonstrated that IL-1R regulates its signaling activity via structural dynamics (Ge et al., 2019). Most IL-1 family members are commonly expressed as full-length precursors that require proteolytic processing for biologically mature forms, leading to significant structural variability. IL-1α is cleaved by the cysteine protease calpain, whereas IL-1β and IL-18 require proteolytic cleavage by the inflammasome (Black et al., 1988; van de Veerdonk et al., 2011). IL-33 and IL-36 utilize neutrophil proteinases such as elastase and proteinase-3 (Clancy et al., 2018), while IL-37 is cleaved by capsase-1 (Kumar et al., 2002) and IL-38 is bioactive as a full-length molecule. Mechanisms between members of the IL-1 family differ in binding interactions with the receptor machinery as Günther et al. have shown that IL-1β and IL-33 use different molecular mechanisms for engaging with the IL-1 receptor accessory protein (IL-1Ra) (Günther et al., 2017). While Hou et al*.* took an alternate approach by leveraging different binding characteristics of IL-1β and IL-1Ra to engineer chimeric receptor antagonists of IL-1R to alleviate the chronically inflammatory dry eye disease (Hou et al., 2013).

Several groups have explored the biophysical aspects of these structures in order to understand how IL-1s transduce signals throughout the cell. One example is the computational approach taken by Ozbabakan et al to map the structural postures of IL-1, IL-1R1 and IL-1RAP as well as downstream signaling proteins (such as MYD88 and TOLLIP) when previously identified oncogenic SNPs cause changes in amino acid residues (Acuner Ozbabacan et al., 2014). This study highlights the change in protein dynamics and its subsequent effect on an entire downstream signaling pathway. The authors present a computationally derived structural pathway detailing the conformational changes related to IL-1 signaling, revealing the mechanisms behind mutations that lead to oncogenic consequences. This work is one example that underscores the importance of atomistic shifts in cytokine structure (rather than a global ensemble) as valuable in the pursuit of targeted therapies and explorations of regulatory mechanisms.

## Case Study IV: Interleukin-2

IL-2 is produced predominantly by activated T-cells in secondary lymphoid organs, where it is consumed by these and other CD25^+^ cells, including regulatory T-cells (T_Reg_) and lymphocytes. IL-2 has been used in both agonistic and antagonistic contexts for immunotherapies, including 1) low-dose efforts to increase T_Reg_ cell counts in autoimmunity, chronic inflammatory conditions and graft rejection (Tahvildari and Dana, 2019) and 2) high-dose administration to expand cytotoxic lymphocyte populations for the treatment of metastatic cancer (Jiang et al., 2016; Buchbinder et al., 2019). Efforts to understand the signaling mechanism and therapeutic potential of IL-2 have focused primarily on cellular and live animal studies, though others have tackled questions relating to its molecular structure and conformational landscape.

**FIGURE 2:**
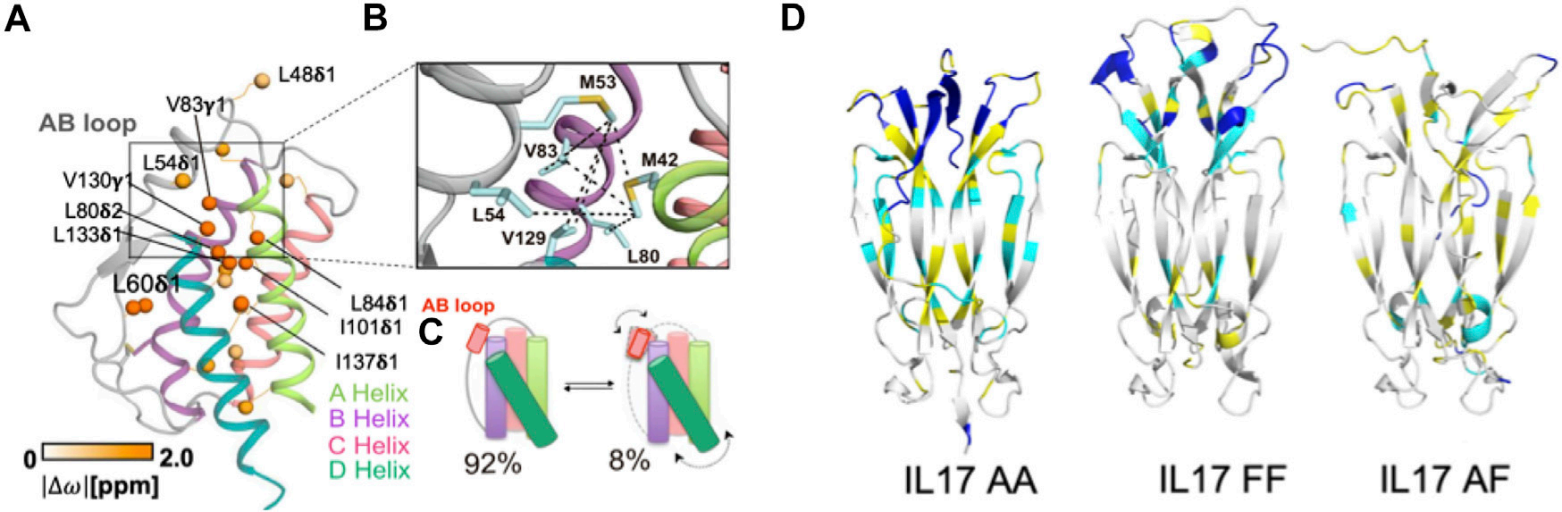
Molecular insights from structure-function studies of interleukins. **(A)** Methyl side chains probed at carbons *via* C (Conlon et al., 2019) labeling in the core of IL-2 undergoing chemical exchange are shown as orange spheres. **(B)** Close-up view of the AB loop showing a network (dotted lines) of observed NOEs in the IL-2 ground state, where the AB loop is well-packed against the hydrophobic core. **(C)** Schematic representation of IL-2 interconversion between ground state (92% population) and excited state (8% populated). Arrows represent concerted conformational changes in the AB loop and hydrophobic core. Figures 2A–C is reproduced with small orientational modifications done in Procreate and PowerPoint from De Paula et al. (2020). **(D)** Cartoon representations of IL-17 in dimeric states, highlighting altered regions of dynamics. ^15^N -^1^H HSQC NMR spectra collected of each IL-17 dimer were compared and regions with differences in backbone amide peak intensities were mapped onto the structure. Residues highlighted in cyan display significantly decreased peak intensities or give rise to double resonances, suggesting highly dynamic sites. Residues highlighted in yellow are amino acids for which peak intensity could not be determined based on spectral overlap, while residues highlighted in blue are unassigned. Figure 2D is reprinted from Cytokine vol. 142, Waters et al., “Conformational dynamics in interleukin 17A and 17F functional complexes is a key determinant of receptor A affinity and specificity.” 2021. With permission from Elsevier.

Critically, it has been established that IL-2 adopts discrete structures in solution that are populated *via* allosteric activation as shown by Sgourakis and coworkers (De Paula et al., 2020). These results build upon a previous body of literature that clearly demonstrate divergent cell fates based on signaling related to the structural plasticity of IL-2. Specifically, methyl-based chemical exchange NMR spectroscopy interrogated IL-2 dynamics on a timescale (*μs-ms*) relevant to enzyme catalysis, ligand binding, and allostery (Figures 2A,B) (Lisi and Loria, 2016). The authors reported that a flexible loop between two N-terminal α-helices acts as a switch (Figure 2C), toggling between an autoinhibited state that has greater affinity for antibody binding, leading to proliferation of T_reg_ cell populations, and a productive state, where IL-2 is primed to interact with IL-2 receptor-α, leading to activation of T_eff_ (effector) cell populations (De Paula et al., 2020). De Paula et al*.* go on to propose an allosteric network of residues serving as the link between the hydrophobic core of IL-2 and the AB loop (Figure 2B), highlighting how a focus on localized molecular motions can lead to novel and detailed mechanistic insight about a cytokine that can be both leveraged and accounted for in future therapeutic efforts.

## Case Study V: Interleukin-17

IL-17 promotes inflammation and plays a protective role in antimicrobial immunity (McGeachy et al., 2019). In the latter case, IL-17 was shown to mediate protection against extracellular pathogens (Valeri and Raffatellu, 2016; Eyerich et al., 2017) by working with IL-22 (a related cytokine also produced by IL-17–expressing cells) to stimulate production of antimicrobial peptides (Wozniak et al., 2014; Mulcahy et al., 2016; Moyat et al., 2017). IL-17 is therefore a double-edged sword with biological properties that make it difficult to predict its role in inflammatory diseases with a polymicrobial etiology. It is possible that IL-17 exerts both protective and destructive effects, as suggested in distinct mouse models (Borkner et al., 2021; Milovanovic et al., 2020; Cruz et al., 2010; Dallenbach et al., 2015; Righetti et al., 2018; O'Connor Jr et al., 2009), although chronic IL-17 receptor signaling can turn a potentially protective acute inflammatory response into chronic immunopathology (Zenobia and Hajishengallis, 2015).

Despite its status as one of best studied cytokines in immunology, recent structure-function relationships and dynamic studies have provided new insight into its regulatory mechanism. Specifically, IL-17 conformational dynamics were shown to dictate signaling efficiency *via* a strong influence over binding affinity for this system. The structural interconversions of IL-17A and IL-17F were studied with NMR spectroscopy by Waters et al, where the IL-17A homodimer was demonstrated to be far more dynamic on slow timescales than the IL-17F homodimer or IL-17A/IL-17F heterodimer (Figure 2D). The aforementioned dynamics aid the IL-17A homodimer in toggling between at least two distinct conformations and the structural perturbations during this process were mapped to the receptor binding region (Waters et al., 2021). The authors propose that these structural fluctuations are likely to affect binding affinity to receptor A (but not other IL-17 receptors), providing insight into the activation of receptor signaling based on atomic level dynamics that cannot be resolved from systems level observations examining only the presence and quantity of IL-17.

## Concluding Remarks

The mechanisms by which cytokines influence a large number of biochemical pathways and molecular interactions have been mysterious. The fact that most proteins in this class have very small structures with simple folds only adds to this paradox. It is difficult to link all of cytokine biochemistry with a common thread, primarily because the majority of information regarding cytokine functions comes from cellular or live animal studies, with comparatively few biophysical investigations. However, the current literature describing cytokine structure has generally shown that 1) protein dynamics that toggle active conformations of cytokines are a driver of functional promiscuity, 2) the cellular or chemical environment modulates cytokine structure and interactions with specific binding partners, and 3) despite their small size, mechanisms of cytokine regulation can be driven by principles of allostery and concerted motion.
